# Supplementary material for: Structure of cryptophyte photosystem II–light-harvesting antennae supercomplex
Source: Nat Commun. 2024 Jun 12;15:4999. doi: 10.1038/s41467-024-49453-0 (PMC11169493; doi:10.1038/s41467-024-49453-0)
Supplement: Supplementary file 1 — Suplementary Information [file 41467_2024_49453_MOESM1_ESM.pdf]

Supplementary Information

for

Zhang et al., Structure of cryptophyte photosystem II–light-harvesting antennae supercomplex

Table S1. Cryo-EM data collection, refinement, and validation statistics.

	**PSII–ACPII (EMDB-38596) (PDB-8XR6)**
**Data Collection and Processing**	
Voltage (kV)	300
Electron exposure (e- /Å^2)	50
Defocus range (um)	-1.2~-2.2
Pixel size (Å)	0.53
Symmetry imposed	C2
Initial particle images (no.)	1,264,152
Final particle images (no.)	168,683
Map resolution (Å)	2.53
FSC threshold	0.143
**Refinement**	
Initial model used (PDB code)	6JLU
Model resolution (Å)	2.53
FSC threshold	0.143
Map sharpening *B* factor (Å^2)	-75
Model composition	
Non-hydrogen atoms	81880
Protein residues	8180
Ligands	378
B factors (Å^2)	
Protein	41.40
Ligand	39.59
R.m.s. deviations	
Bond lengths (Å)	0.009
Bond angles (º)	1.266
**Validation**	
MolProbity score	1.49
Clashscore	5.97
Poor rotamers (%)	0.17
Map VS model	
CC_{mask}	0.83
CC_{volume}	0.84
CC_{peak}	0.80
**Ramachandran plot**	
Favored (%)	97.06
Allowed (%)	2.91
Outliers (%)	0.02

Table S2. Comparison of the subunits in PSII–LHCII of red algae, cryptophyte, and diatoms.

Subunit	Red algae	Cryptophyte	Diatoms
PsbA (D1)	√	√	√
PsbB (CP47)	√	√	√
PsbC (CP43)	√	√	√
PsaD (D2)	√	√	√
PsbE	√	√	√
PsbF	√	√	√
PsbG			√
PsbH	√	√	√
PsbI	√	√	√
PsbJ	√	√	√
PsbK	√	√	√
PsbL	√	√	√
PsbM	√	√	√
PsbO	√	√	√
PsbP			
PsbQ’	√	√	√
PsbR			
PsbS			
PsbT	√	√	√
PsbU	√	√	√
PsbV	√	√	√
PsbW	√	√	√
PsbX	√	√	√
PsbY	√		
PsbZ	√	√	√
Ycf12/Psb30	√	√	√
Psb31	√		√
Psb34	√		√
Psb-γ		√	
LHCII	0	12	12/22

Table S3. Cofactors in each subunit of the cryptophyte PSII–ACPII structure.

Subunit	Chain	Traced residues	Chls	Cars	Lipids	Others
PsbA	A, a	334 (11-344)	3 a, 1 pheophytin	1 α-Car	2 SQDG, 1 LHG, 1 LMG	1 Mn_4CaO_5 cluster, 1 PQ, 1 Fe, 1 Cl^-
PsbB	B, b	503 (2-504)	17 a	3 α-Car	1 DGDG, 1 SQDG, 3 LMG, 2 LHG	
PsbC	C, c	80 (23-473)	13 a	3 α-Car	2 DGDG, 1 LMG	
PsbD	D, d	342 (10-351)	3 a, 1 pheophytin	1 α-Car	1 LHG, 3 LMG	1 PQ, 1 HCO_3^-
PsbE	E, e	75 (9-83)				
PsbF	F, f	29 (14-42)				1 Heme
PsbH	H, h	65 (2-66)		1 α-Car		
PsbI	I, i	35 (1-35)				
PsbJ	J, j	34 (6-39)			1 DGDG, 1 SQDG	
PsbK	K, k	37 (9-45)		1 α-Car		
PsbL	L, l	37 (2-38)				
PsbM	M, m	37 (1-37)				
PsbO	O, o	262 (67-328)				
PsbQ’	Q, q	143 (59-201)				
PsbT	T, t	31 (1-31)				
PsbU	U, u	93 (29-121)				
PsbV	V, v	137 (27-163)				1 Heme
PsbW	W, w	49 (43-91)				
PsbX	X, x	39 (1-39)				
PsbZ	Z, z	61 (1-61)				
Psb30	Y, y	34 (1-34)				
Psb-γ	N, n	190 (64-253)	2 a			
ACPII-1	1, 7	175 (43-217)	9 a, 2 c	4 Alx, 1 α-Car	1 LHG	
ACPII-2	2, 8	187 (40-226)	11 a, 1 c	4 Alx, 1 Cro	2 LHG	
ACPII-3	3, 9	185 (42-226)	12 a, 1 c	4 Alx, 1 Cro	2 LHG	
ACPII-4	4, 0	176 (42-218)	10 a, 1 c	4 Alx, 1 α-Car	2 LHG	
ACPII-5	5, g	170 (43-212)	13 a, 1 c	4 Alx, 1 Cro	1 LHG	
ACPII-6	6, p	192 (40-231)	12 a, 1 c	4 Alx, 1 Cro		
Total			209 a, 14 c, 4 pheophytin	48 Alx, 24 α-Car, 8 Cro	8 DGDG, 8 SQDG，24 LHG, 16 LMG	2 Mn_4CaO_5 cluster, 4 PQ, 2 HCO_3^-, 4 Heme, 2 Fe, 2 Cl^-

SQDG, sulfoquinovosyldiacyl glycerol; LMG, distearoylmonogalactosyl diglyceride; DGDG, digalactosyldiacyl glycerol; LHG, dipalmitoylphosphatidyl glycerol; a, chlorophyll *a*; c, chlorophyll *c*; Alx, alloxanthin; Cro, crocoxanthin; α-Car, α-carotene; PQ, plastoquinone.

Table S4. Pigment-binding sites in the 6 ACPIIs.

Sites	ACPII-1	ACPII-2	ACPII-3	ACPII-4	ACPII-5	ACPII-6
301	Chl *a*	Chl *a*	Chl *a*	Chl *a*	Chl *a*	Chl *a*
302	Chl *a*	Chl *a*	Chl *a*	Chl *a*	Chl *a*	Chl *a*
303	Chl *c*	Chl *c*	Chl *c*	Chl *a*	Chl *c*	Chl *c*
304	Chl *a*	Chl *a*	Chl *a*	Chl *a*	Chl *a*	Chl *a*
305	Chl *a*	Chl *a*	Chl *a*	Chl *a*	Chl *a*	Chl *a*
306	Chl *a*	Chl *a*	Chl *a*	Chl *a*	Chl *a*	Chl *a*
307	Chl *a*	Chl *a*	Chl *a*	Chl *a*	Chl *a*	Chl *a*
308	Chl *a*	Chl *a*	Chl *a*	Chl *a*	Chl *a*	Chl *a*
309	Chl *a*	Chl *a*	Chl *a*	Chl *a*	Chl *a*	Chl *a*
310	Chl *c*	Chl *a*	Chl *a*	Chl *c*	Chl *a*	Chl *a*
311		Chl *a*	Chl *a*		Chl *a*	Chl *a*
312	Chl *a*	Chl *a*	Chl *a*	Chl *a*	Chl *a*	Chl *a*
313			Chl *a*			
314					Chl *a*	
315					Chl *a*	
316						Chl *a*
401	Alx	Alx	Alx	Alx	Alx	Alx
402	Alx	Alx	Alx	Alx	Alx	Alx
403	Alx	Alx	Alx	Alx	Alx	Alx
404	α-Car	Cro	Cro	α-Car	Cro	Cro
405	Alx	Alx	Alx	Alx	Alx	Alx

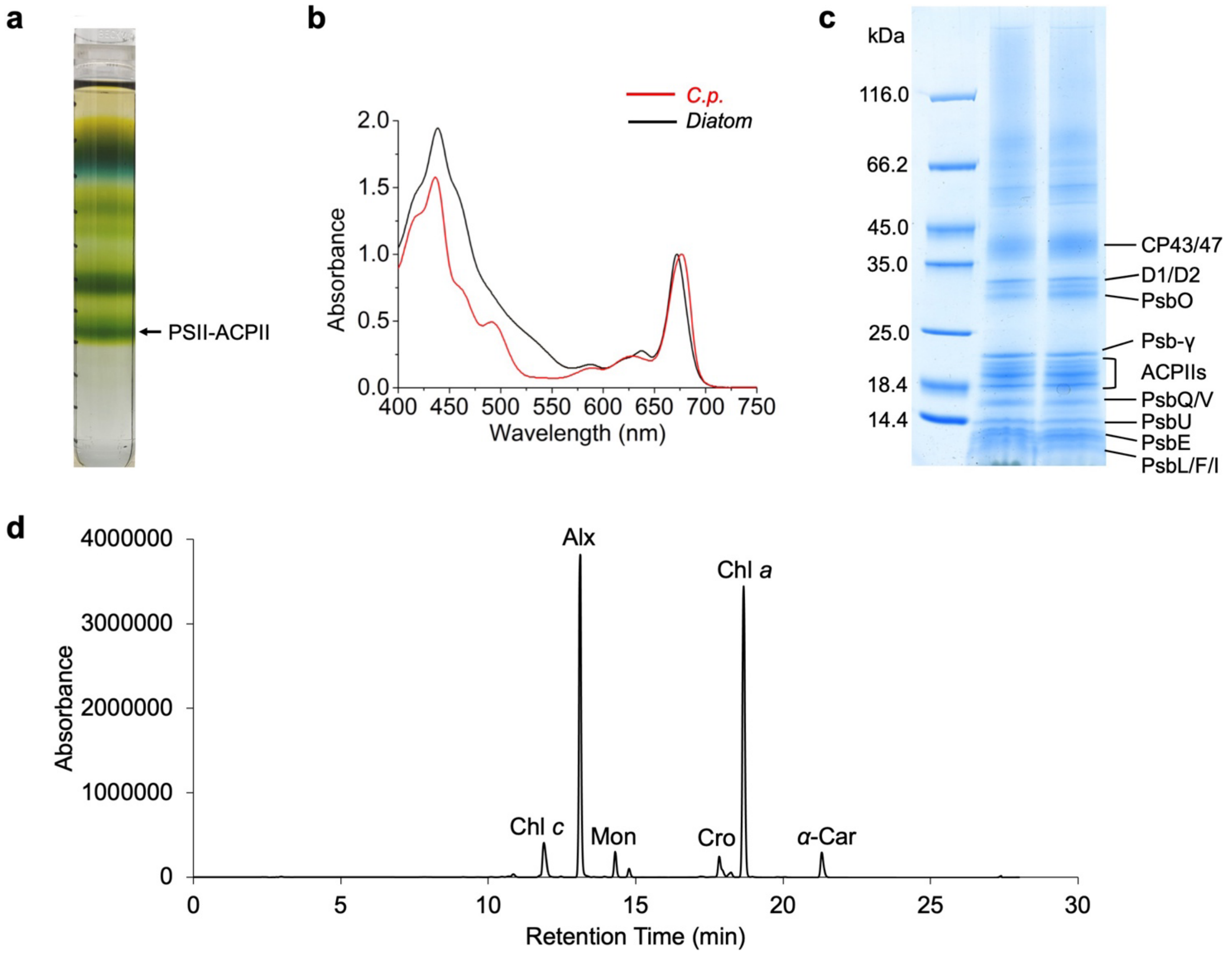

Fig. S1. Preparation and characterization of PSII–ACPII from *Chroomonas placoidea*. **a**, Isolation of the PSII–ACPII supercomplex by using sucrose density gradient ultracentrifugation. **b**, Room-temperature absorption spectra of the *Chroomonas placoidea* (*C.p.*) PSII–ACPII and diatom *Chaetoceros gracilis* PSII–FCPII supercomplex. **c**, SDS-PAGE analysis of the PSII–ACPII supercomplex. The protein composition of the bands was indicated based on the mass spectrometry analysis. **d**, Analysis of the pigment composition of PSII–ACPII by HPLC, recorded at 445 nm. Based on the characteristic absorption spectrum of each peak fraction, six major pigment peaks were identified as chlorophyll *c* (Chl *c*), alloxanthin (Alx), monadoxanthin (Mon), crocoxanthin (Cro), chlorophyll a (Chl *a*) and *α*-carotene (*α*-Car) respectively. These experiments were performed for more than five times, and the same results were obtained reproducibly. Source data for Supplementary Figs. 1b, 1c, and 1d are provided.

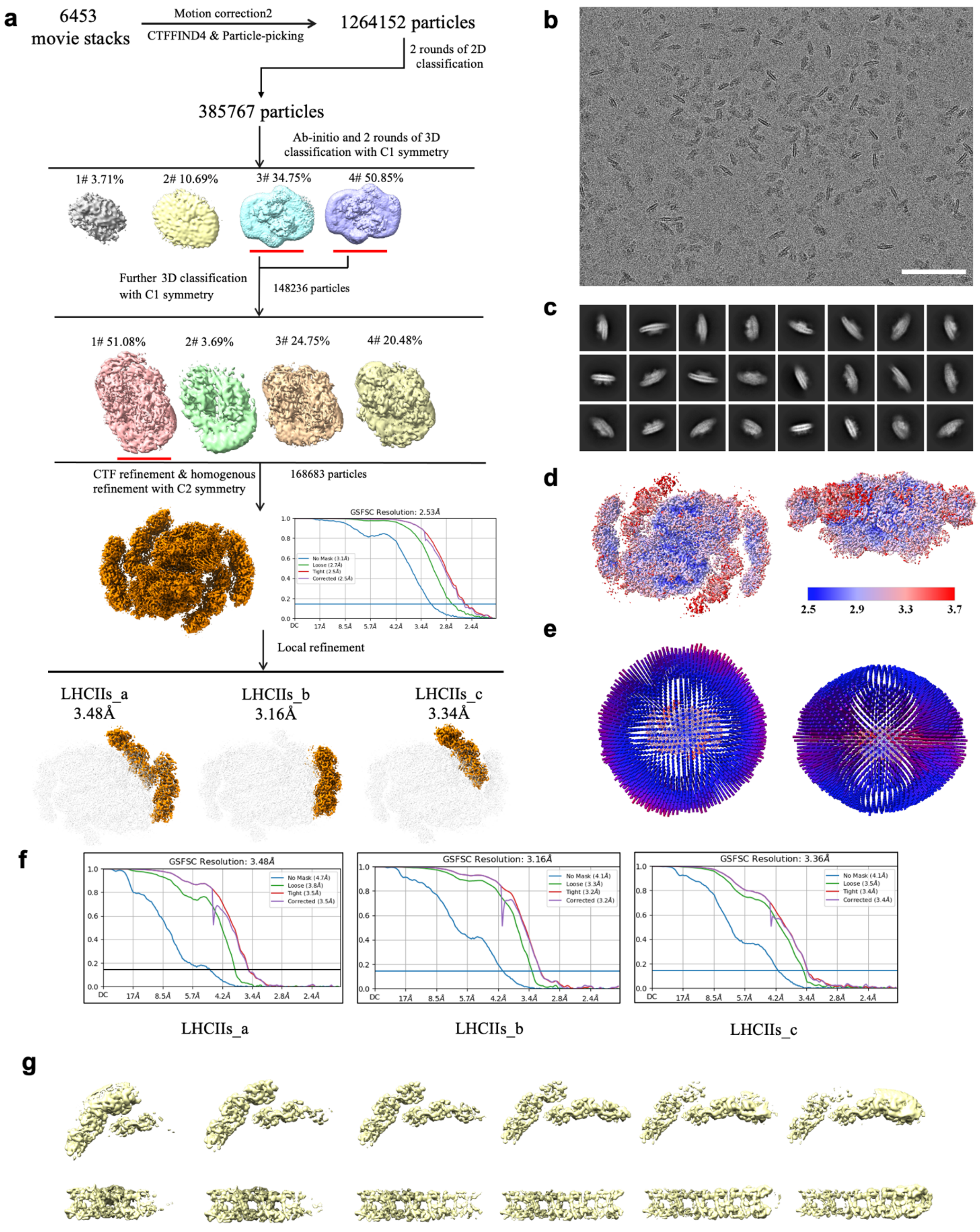

Fig. S2. Cryo-EM data processing for the PSII–ACPII supercomplex. **a**, Schematic flowchart for the cryo-EM data processing. **b**, A representative cryo-EM micrograph of the PSII–ACPII supercomplex. **c**, Representative 2D classes of the PSII–ACPII supercomplex. The box size is 424 Å. **d**, Local resolution distributions of the cryo-EM map estimated by ResMap. **e**, Angular distribution of particles used for the reconstruction of the final density map. **f**, Local resolution estimated by cryoSPARC. Scale bar, 100 nm. **g**, Representative cryo-EM images of the 3D variability analysis of the peripheral subunits of PSII-ACPII. The images below show the perspective obtained by rotating the images above by 90º. Scale bar, 100 nm.

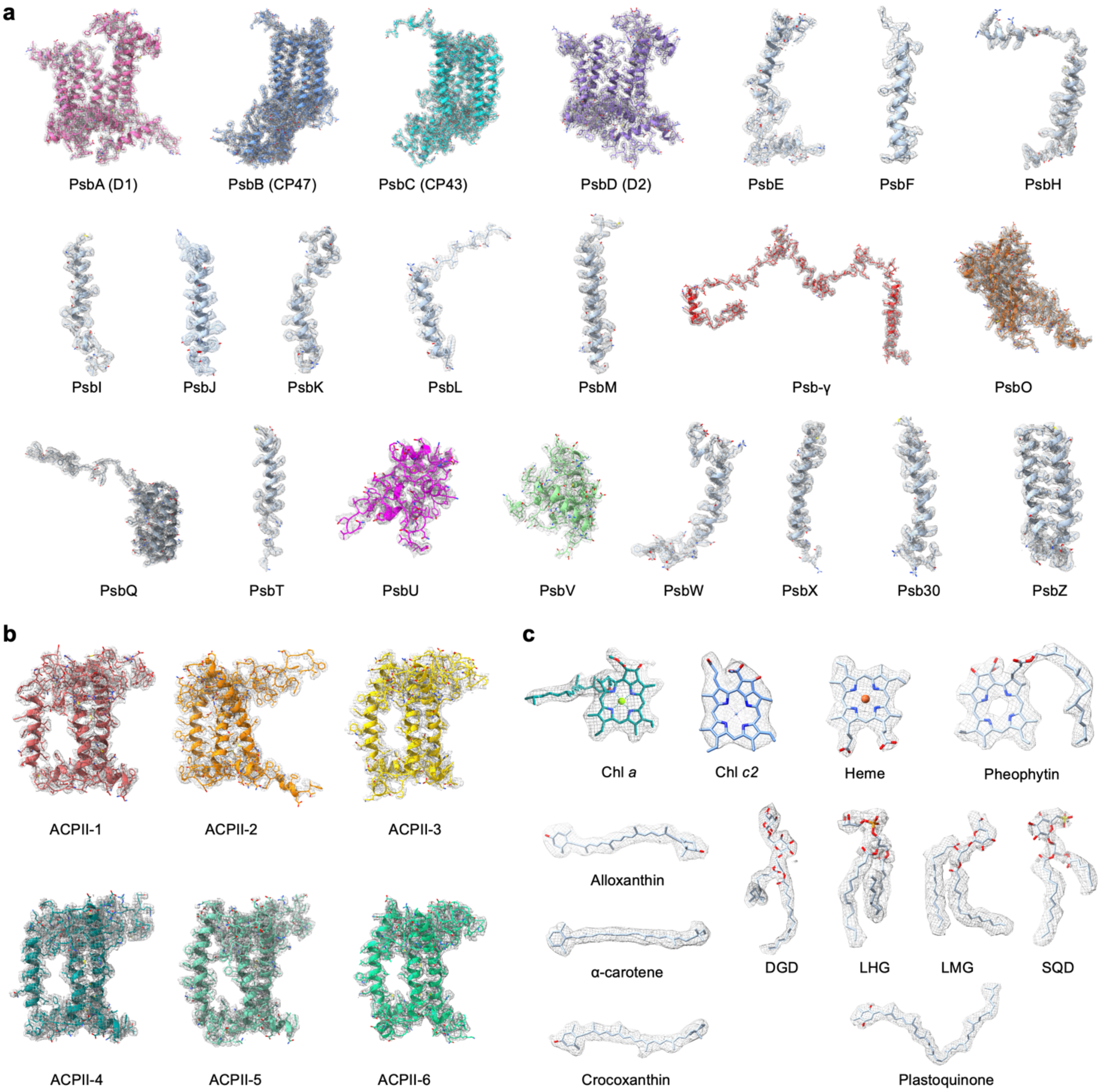

Fig. S3. Cryo-EM density maps and structures of the antennas and pigment molecules. The PSII core and antenna subunits are shown in cartoons, and the cryo-EM density maps of individual subunits and cofactors are depicted in gray meshes. **a**, Cryo-EM densities and structural models of the PSII core subunits. **b**, Cryo-EM density and the structures of six ACPIIs. **c**, Cryo-EM densities of the typical PSII ligands and pigments.

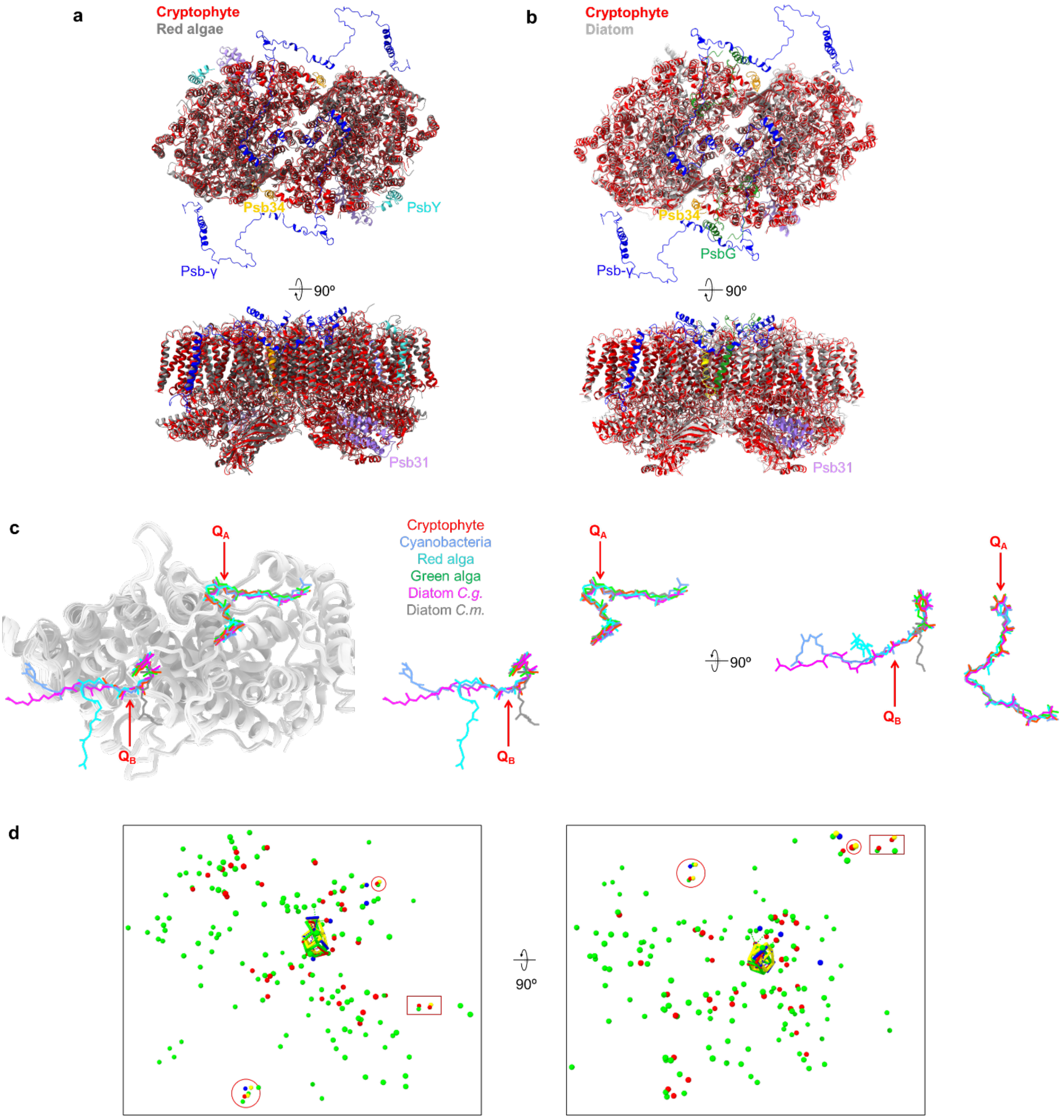

Fig. S4. Comparison of the PSII cores of Cryptophyte with those of other algae. a, Superposition of the cryptophyte PSII core (red) with red algal PSII core (dim gray, PDB: 7Y5E) and diatom PSII core (light gray, PDB: 6JLU). **b**, Psb-γ, which is absent in other PSII cores, is labeled in blue. PsbG, PsbY, Psb31, and Psb34, which are absent in the cryptophyte PSII core, are indicated. **c**, Comparison of the locations and structures of Q_A and Q_B in cryptophyte PSII–ACPII (red) with those in PSII–LHCIIs of the cyanobacterium *Thermostichus vulcanus* (blue, PDB: 8IR5), red alga *Porphyridium purpureum* (cyan, PDB: 7Y5E), the green alga *Chlamydomonas reinhardtii* (green, PDB: 6KAF), diatom *Chaetoceros gracilis* (magenta, PDB: 7VD5), and diatom *Cyclotella meneghiniana* (gray, PDB: 8J5K). **d**, Comparison of the locations and structures of water molecules in cryptophyte PSII (red) with those in PSIIs of the cyanobacterium *T. vulcanus* (green, PDB: 8IR5), red alga *Cyanidium caldarium* (blue, PDB: 4YUU), diatom *C. gracilis* (yellow, PDB: 7VD5). Representative areas have been encircled to illustrate the relative positions between the two images.

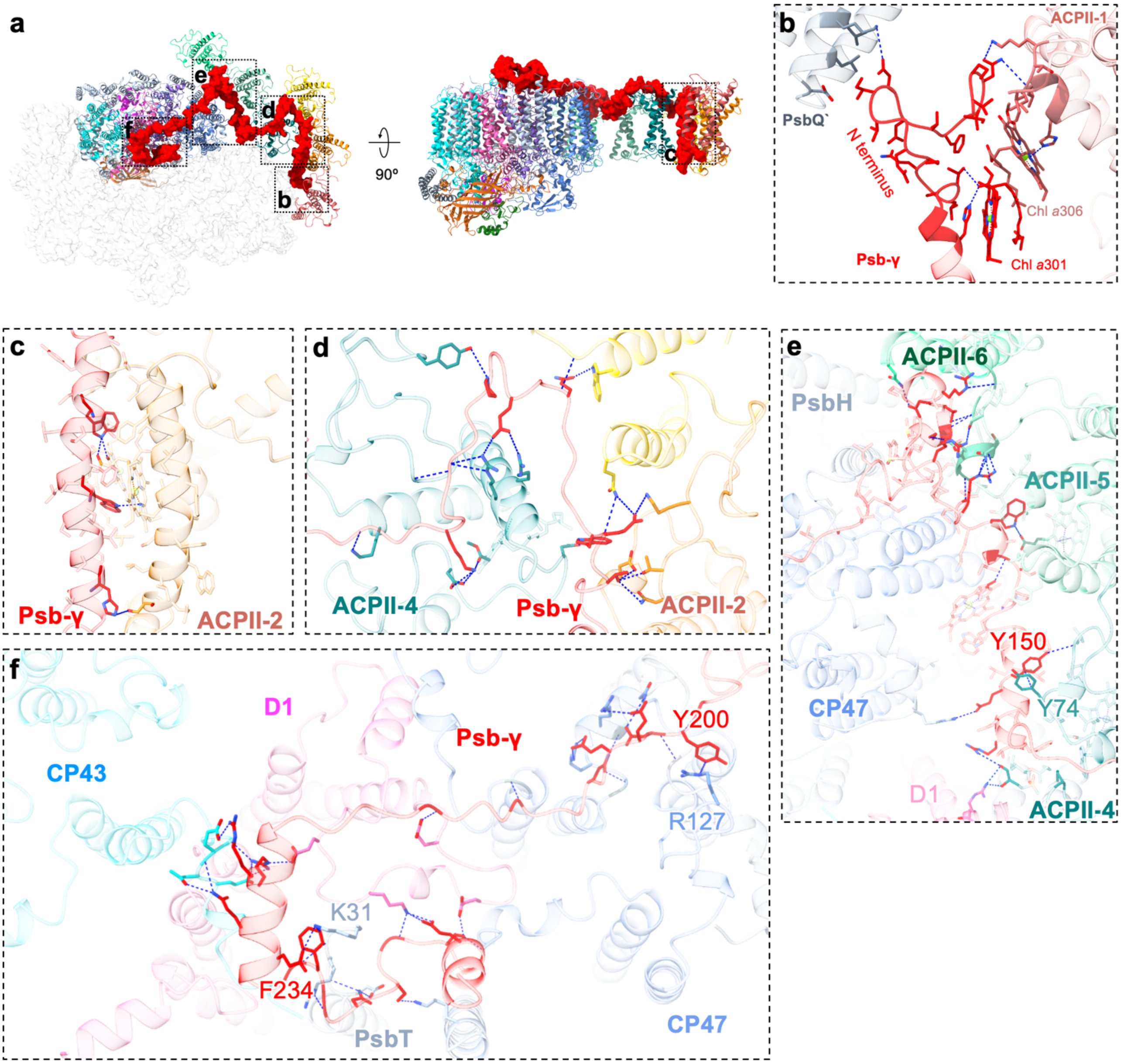

Fig. S5. Psb-γ interacts with the PSII core and ACPIIs. a, Overview of the interactions of Psb-γ within the PSII–ACPII supercomplex. The detailed connections are shown in enlarged boxes. **b**, Interactions of the N-terminus of Psb-γ with PsbQ' and ACPII-1. **c**, Interactions between the TMH parts of Psb-γ and ACPII-2. **d**, Interactions of Psb-γ with ACPII-4, ACPII-3 and ACPII-2. **e**, Interactions of Psb-γ with the PSII core, ACPII-6, ACPII-5, and ACPII-4. **f**, Interactions between the C-terminus of Psb-γ and the PSII core. Hydrogen bonds and salt bridges are indicated by blue dashed lines.

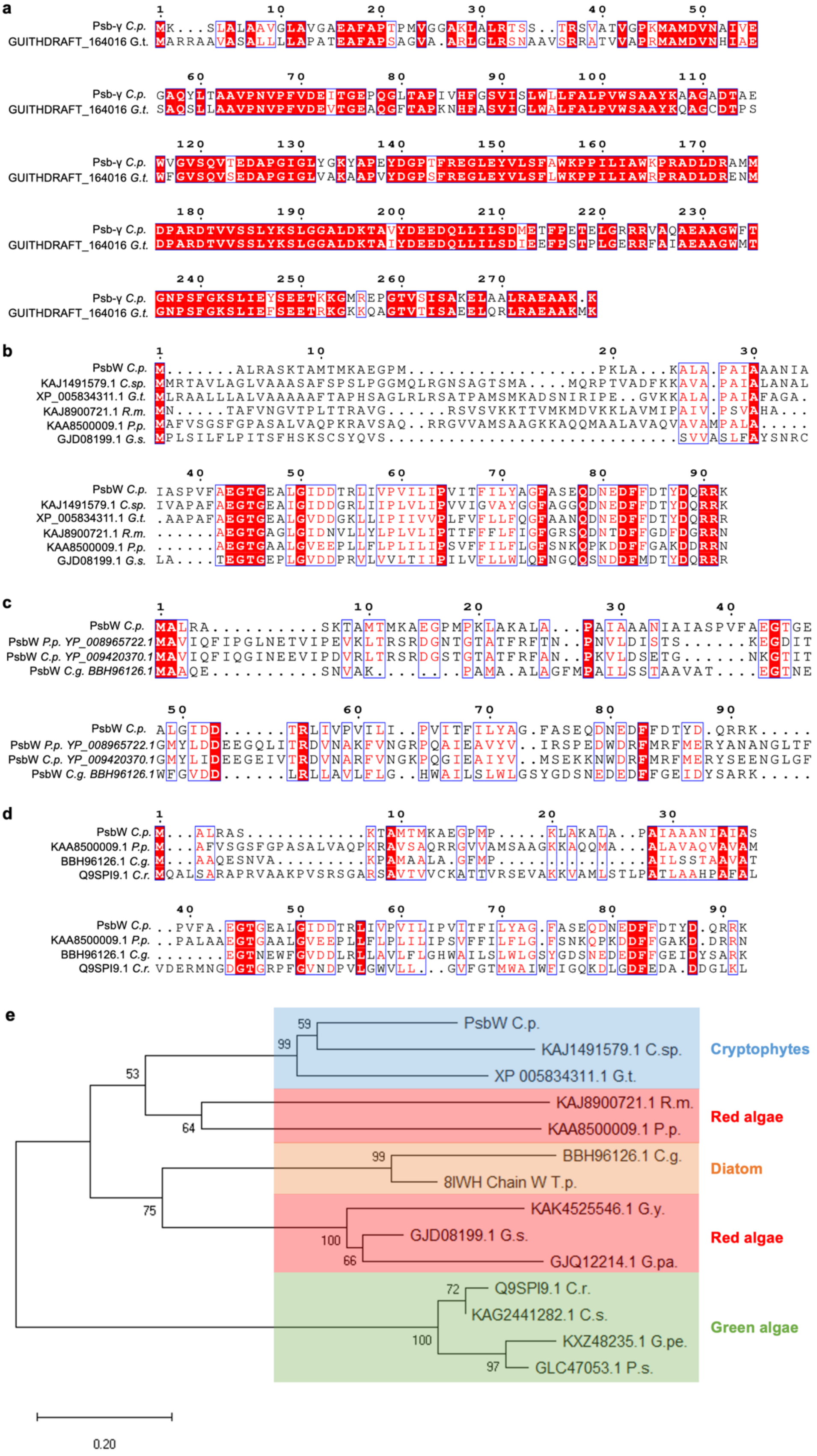

Fig. S6. Sequence alignments of Psb-γ and PsaW of PSII–ACPII and the corresponding sequences from red algae, cryptophytes, diatoms and green algae. a, Comparison of the sequence of Psb-γ from *Chroomonas placoidea* (*C.p.*) and the homologous sequence GUITHDRAFT_164016 from *Guillardia theta* (*G.t.*). **b,** Comparison of the sequence of PsbW from *Chroomonas placoidea* (*C.p.*) in this study and the

homologous sequences from cryptophytes and red algae. *Cryptophyta sp. CCMP2293: C.sp., Guillardia theta CCMP2712: G.t., Rhodosorus marinus: R.m., Porphyridium purpureum: P.p., Galdieria sulphuraria: G.s.*. **c,** Comparison of the sequence of PsbW from *Chroomonas placoidea* (*C.p.*) in this study and sequences of PsbW in National Center of Biotechnology Information from red alga *Porphyridium purpureum* (*P.p.*), *Chroomonas placoidea* (*C.p.*) and diatom *Chaetoceros gracilis* (*C.g.*). **d,** Comparison of the sequence of PsbW from *Chroomonas placoidea* (*C.p.*) in this study and the sequences of PsbW in the PSII structures of red alga *Porphyridium purpureum* (*P.p.*), diatom *Chaetoceros gracilis* (*C.g.*), and green algae *Chlamydomonas reinhardtii* (*C.r.*). **e,** Phylogenetic tree of PsbW from PSII structures of cryptophyte *Chroomonas placoidea* (*C.p.*), red alga *Porphyridium purpureum* (*P.p.*), diatom *Chaetoceros gracilis* (*C.g.*), green algae *Chlamydomonas reinhardtii* (*C.r.*). and their homologous sequences. *Cryptophyta sp. CCMP2293: C.sp., Guillardia theta CCMP2712: G.t., Rhodosorus marinus: R.m., Porphyridium purpureum: P.p., Galdieria yellowstonensis: G.y., Galdieria sulphuraria: G.s. Galdieria partita: G.pa., Chaetoceros gracilis: C.g., Thalassiosira pseudonana: T.p., Chlamydomonas reinhardtii: C.r., Chlamydomonas schloesseri: C.s., Gonium pectoral: G.pe., Pleodorina starrii: P.s.*. The phylogenetic tree was constructed using Neighbor-Joining method based on amino acid sequences. The tree was built with the Poisson model using 133 amino acid residues, and a bootstrap test (1000 replicates) was conducted.

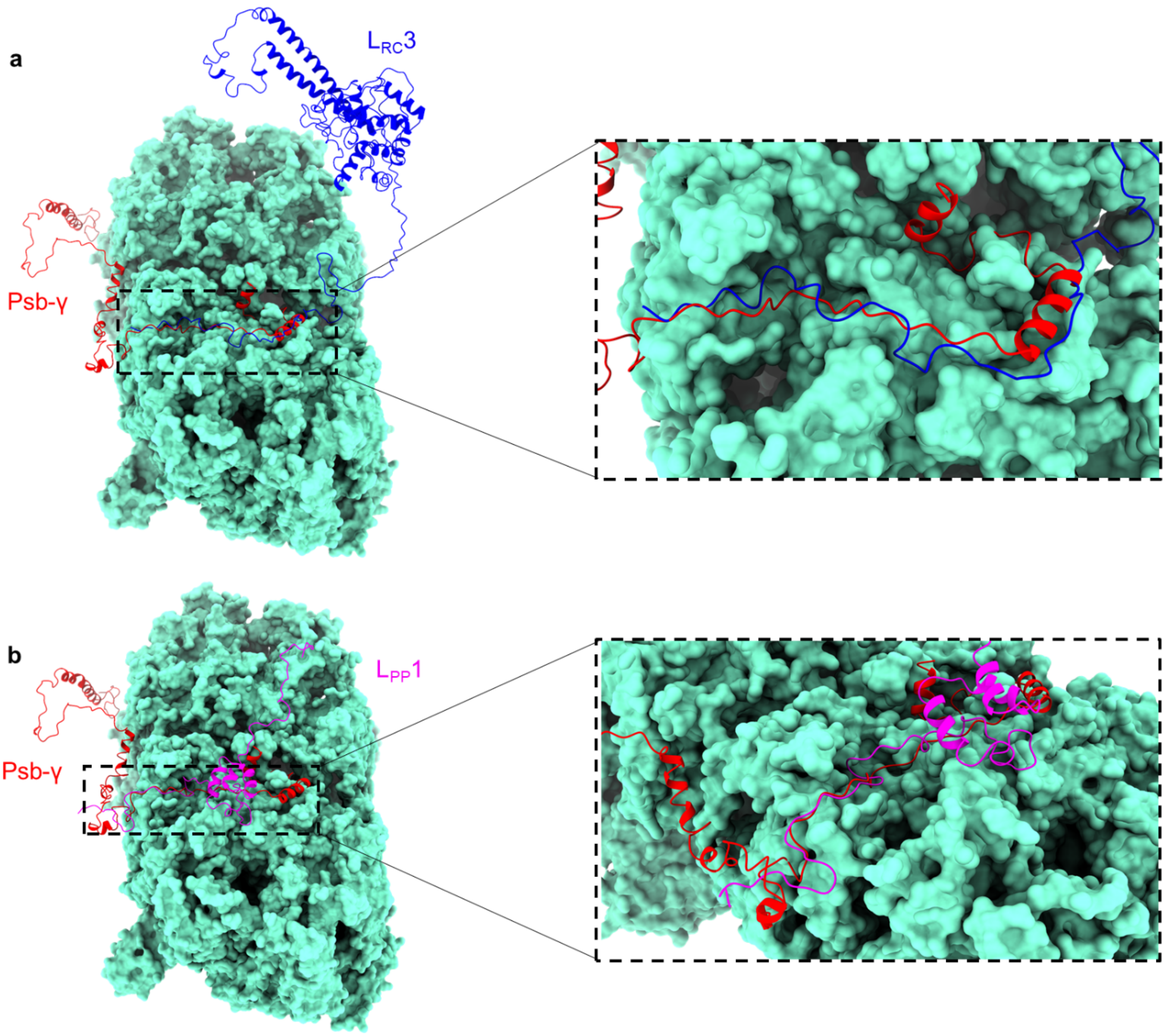

Fig. S7. Comparison of the Psb-γ of cryptophyte with $L_{PP}1$ and $L_{RC}3$ of red algae. The cryptophyte PSII core and red algal PSII core (PDB: 7Y5E) are superimposed. Psb-γ is highlighted in red. **a**, $L_{RC}3$ of red algae is indicated in blue. **b**, $L_{PP}1$ of red algae is indicated in magenta.

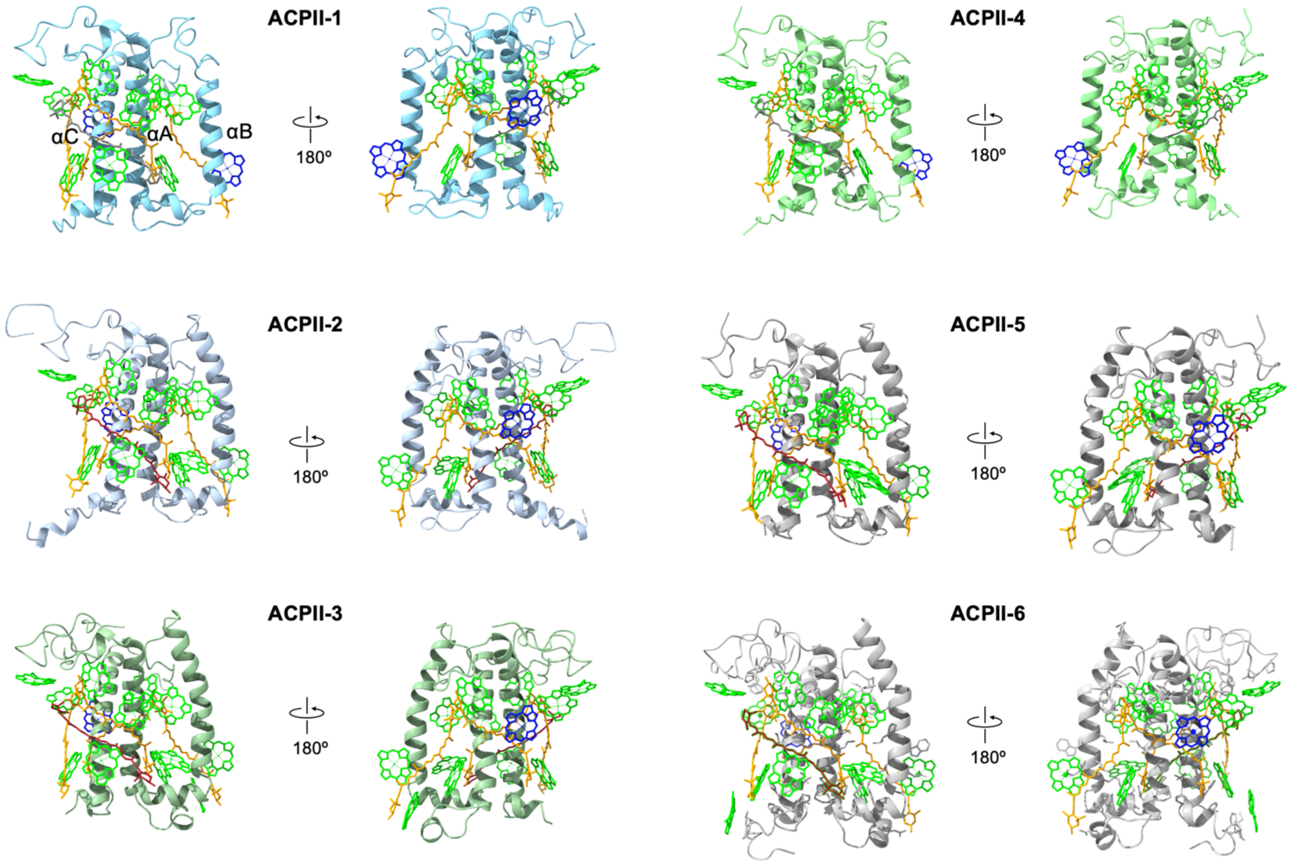

Fig. S8. Structures of individual ACPII subunits. Chl *a*, Chl *c*, alloxanthin, crocoxanthin, monadoxanthin, and α-carotene are colored green, blue, orange, brown, magenta, and gray, respectively, with the phytol chains omitted.

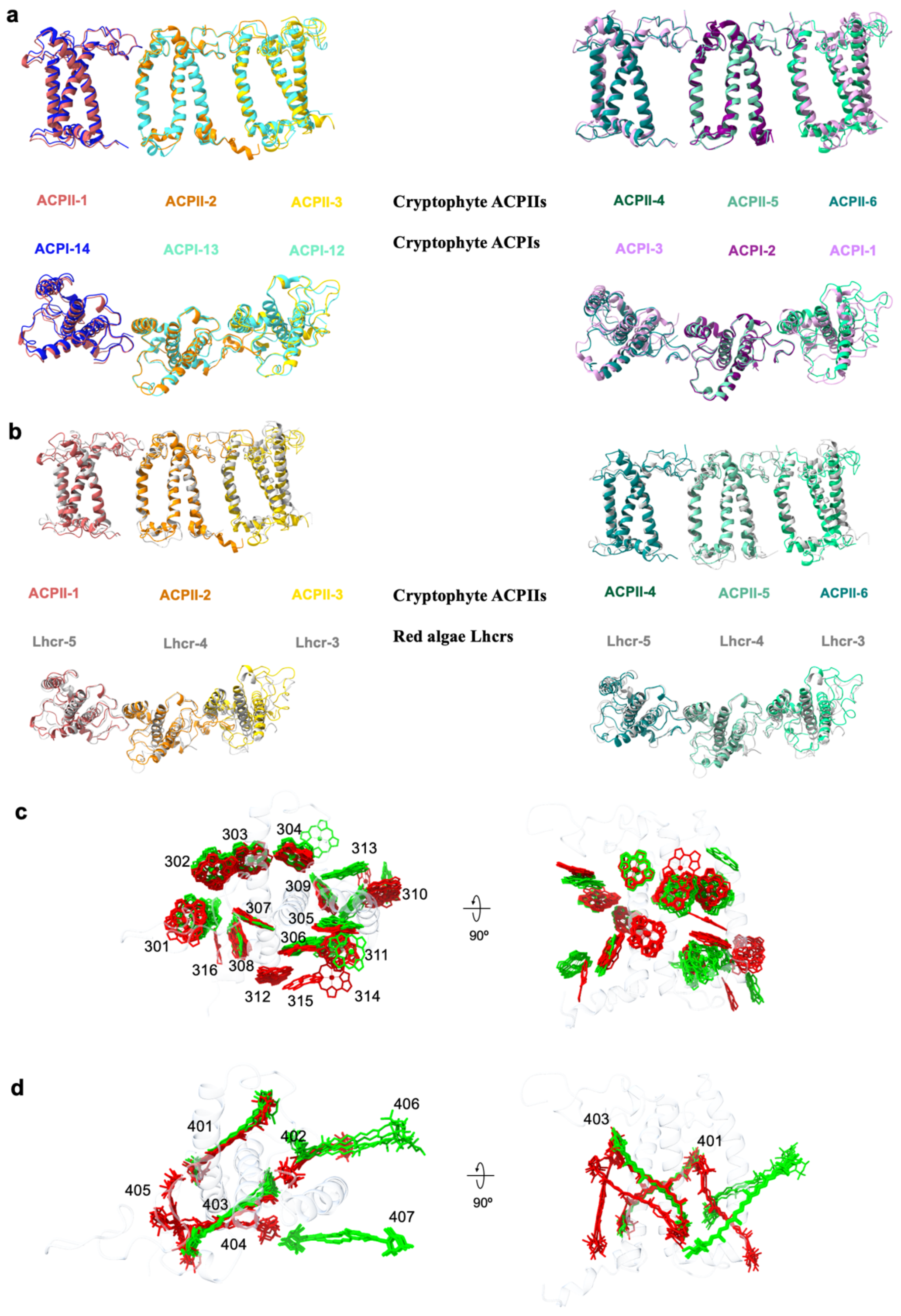

Fig. S9. Structure comparison of cryptophyte ACPIIs with cryptophyte ACPIs and red algal LHCRs and pigment-binding sites comparison of cryptophyte ACPIIs with green algae LHCIIs. a, Superposition of the cryptophyte ACPII-1/2/3, ACPII-4/5/6 with cryptophyte ACPI-14/13/12 and ACPI-3/2/1 (PDB: 7Y7B), respectively. **b**, Superposition of the cryptophyte ACPII-1/2/3, ACPII-4/5/6 with red algal Lhcr-5/4/3 colored gray (PDB: 7Y5E), respectively. ACPI-14 and ACPI-2 share the same protein sequences as ACPII-1 and ACPII-5, which are labeled in blue and purple, respectively. ACPI-12/13 are labeled in cyan, and ACPI-1/3 are labeled in magenta. The colors of the ACPIIs are the same as shown in Fig. 1. **c-d**, Superposition of the chlorophylls (**c**) and carotenoids binding sites (**d**) from cryptophyte ACPIIs (red) and LHCIIs of the green alga *Chlamydomonas reinhardtii* (green, PDB: 6KAF). The LHCIIs include CP26, CP29, and LHCII subunits from the S-, M- and N-LHCII trimers.

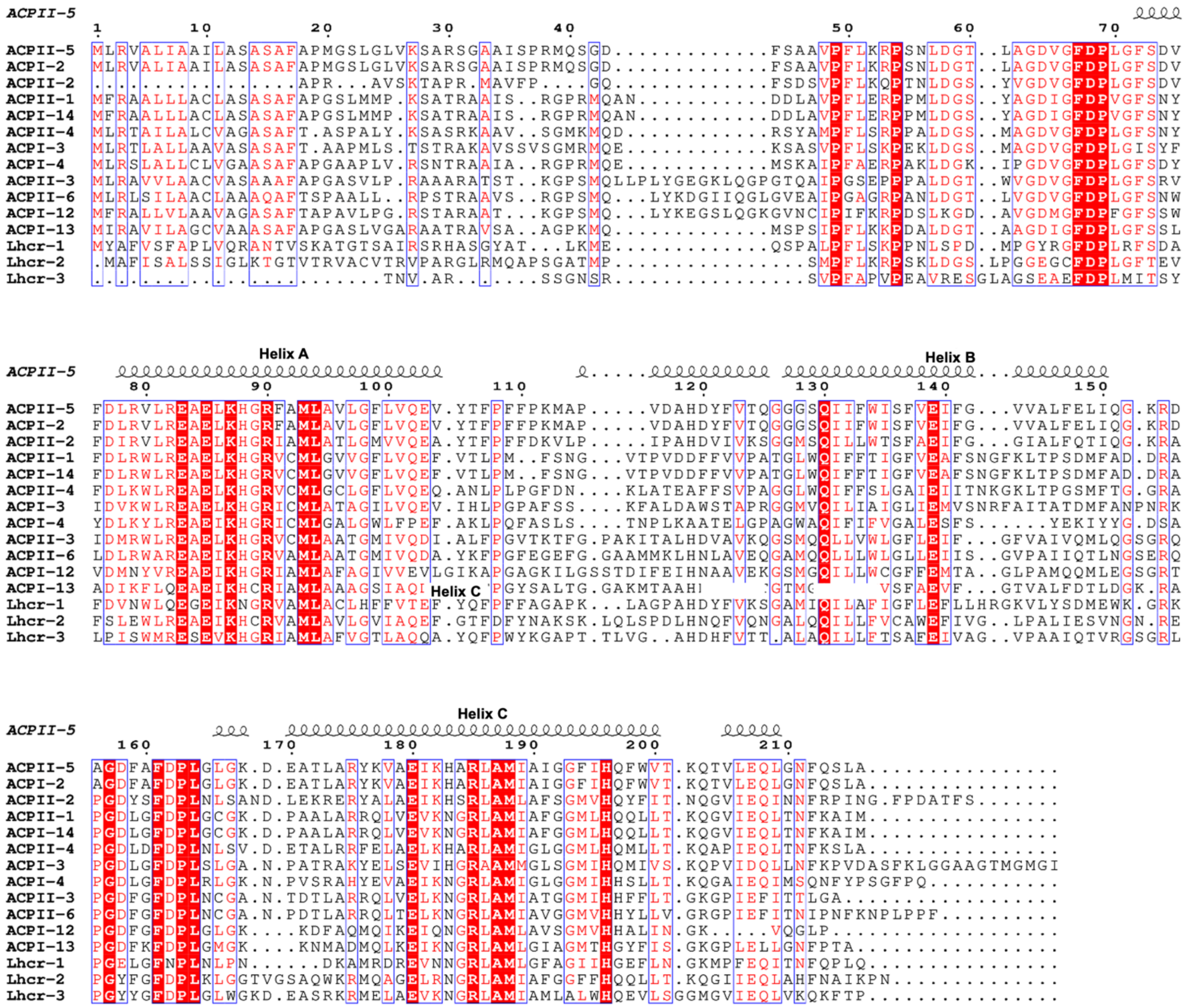

Fig. S10. Sequence alignment of ACPIIs from cryptophyte PSII–ACPII with the corresponding sequences from cryptophyte PSI–ACPI and red algae LHCRs. The secondary structure is shown above the sequences. Fully conserved residues are shaded in red, and similar amino acids are highlighted by blue frames.

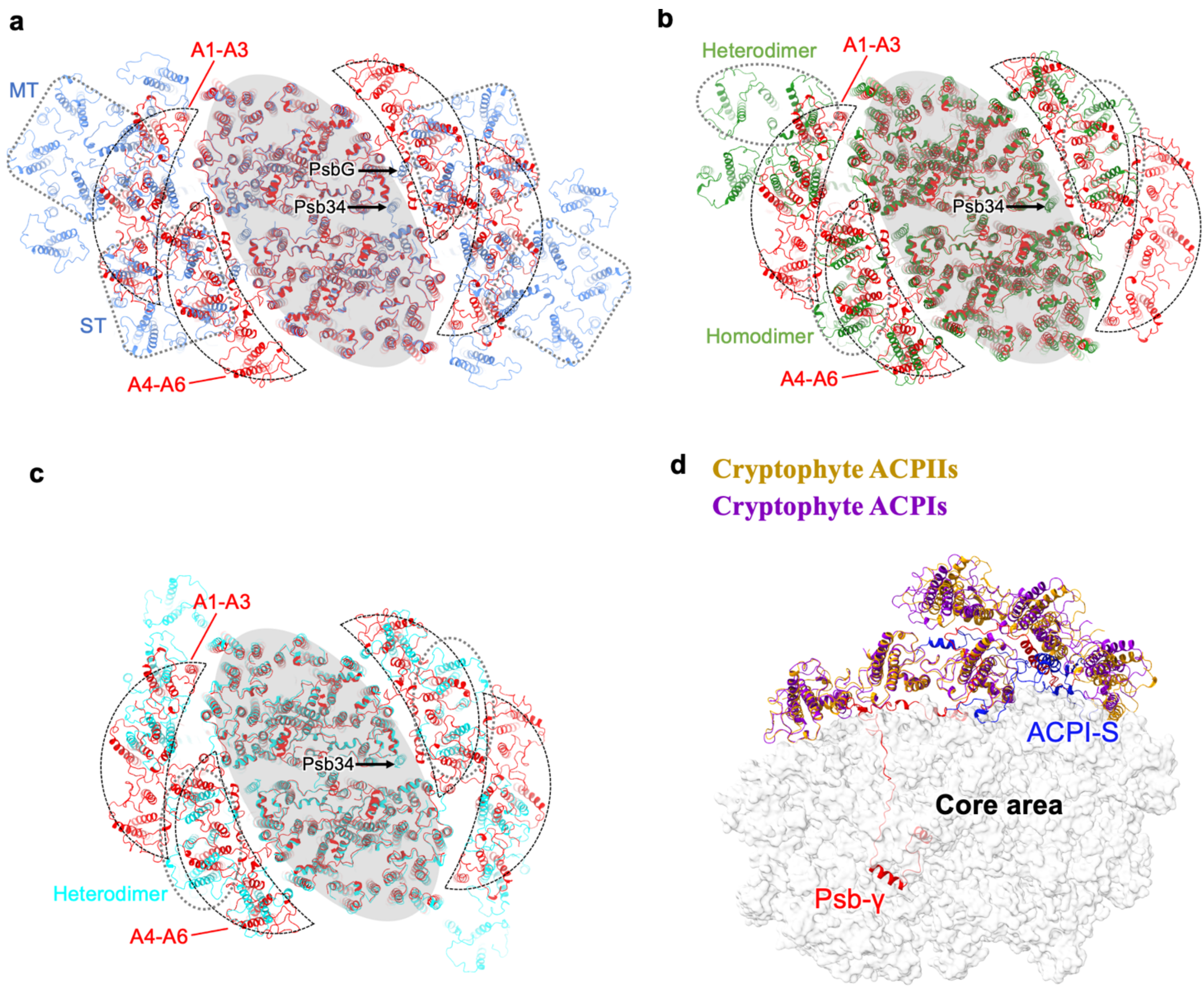

Fig. S11. Comparison of the arrangements of ACPIIs in cryptophyte PSII–ACPII with FCPIIs in diatom PSII–FCPII and ACPIs in cryptophyte PSI–ACPI. a, Superposition of the PSII–ACPII structure (red) with PSII–FCPII (PDB: 7VD5) from diatom *Chaetoceros gracilis* (blue). **b**, Superposition of the PSII–ACPII structure (red) with PSII–FCPII (PDB: 8IWH) from diatom *Thalassiosira pseudonana* (green). **c**, Superposition of the PSII–ACPII structure (red) with PSII–FCPII (PDB: 8J5K) from diatom *Cyclotella meneghiniana* (cyan). A1-A3 trimer and A4-A6 trimer of cryptophyte are labeled. Letters of A1-A6 represent ACPII1-6. Strongly associated LHCII tetramer (ST), moderately associated LHCII tetramer (MT), and LHCII dimers of diatom are indicated (tetramer: gray dashed box, dimer: gray dashed oval). **d**, Superposition of the PSII–ACPII structure (orange) with PSI–ACPI (PDB: 7Y7B) from cryptophyte (cyan). The linker protein ACPI-S is colored blue and Psb-γ is colored red.

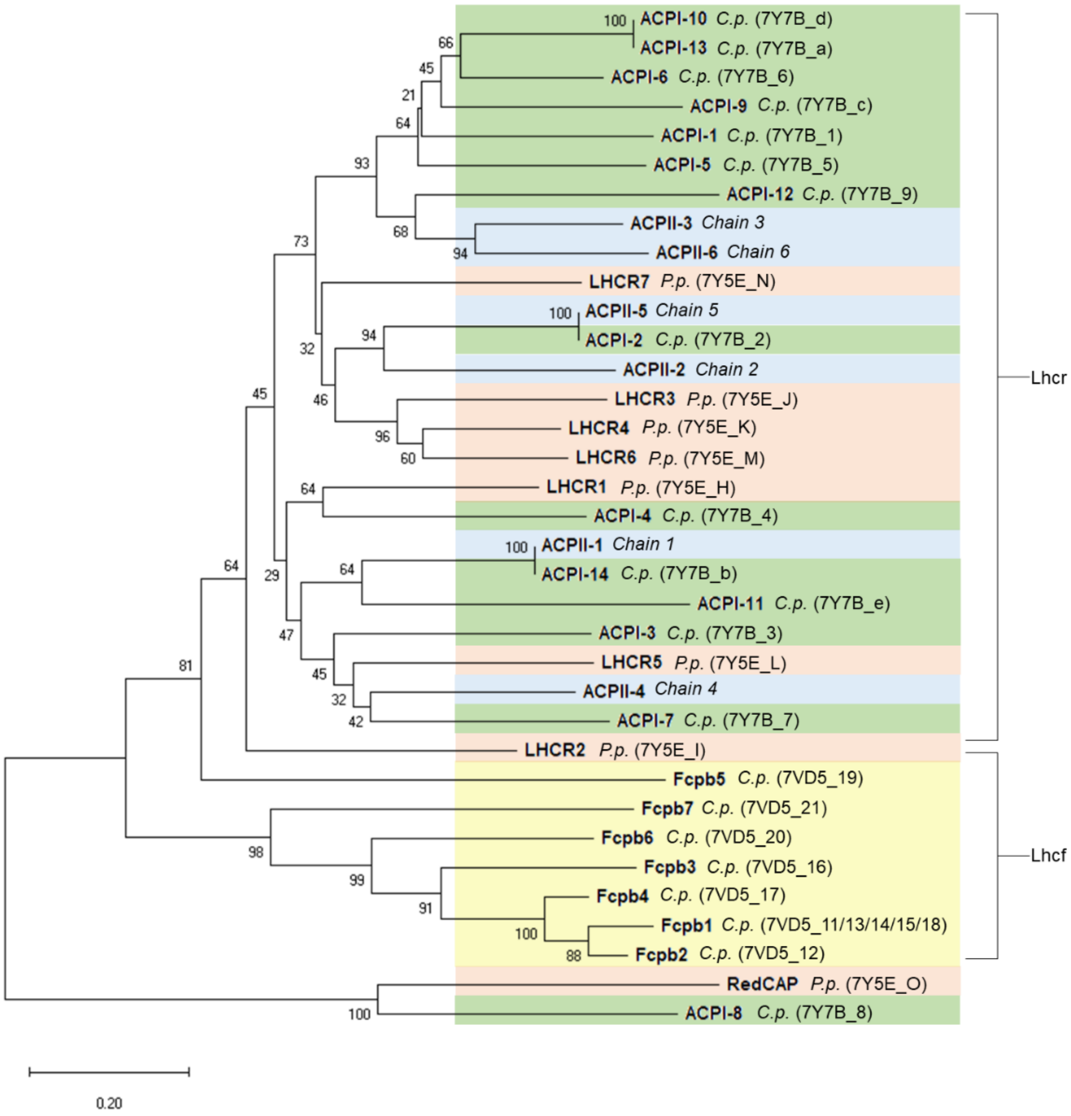

Fig. S12. Phylogenetic tree of LHCIIs in cryptophytic alga *C. placoidea* (C.p.), LHCIIs in diatom *C. gracilis* (C.g.) and LHCIs in red alga *P. purpureum* (P.p.). The phylogenetic tree was constructed using Neighbor-Joining method based on amino acid sequences. The tree was built with the Poisson model using 327 amino acid residues, and a bootstrap test (1000 replicates) was conducted.

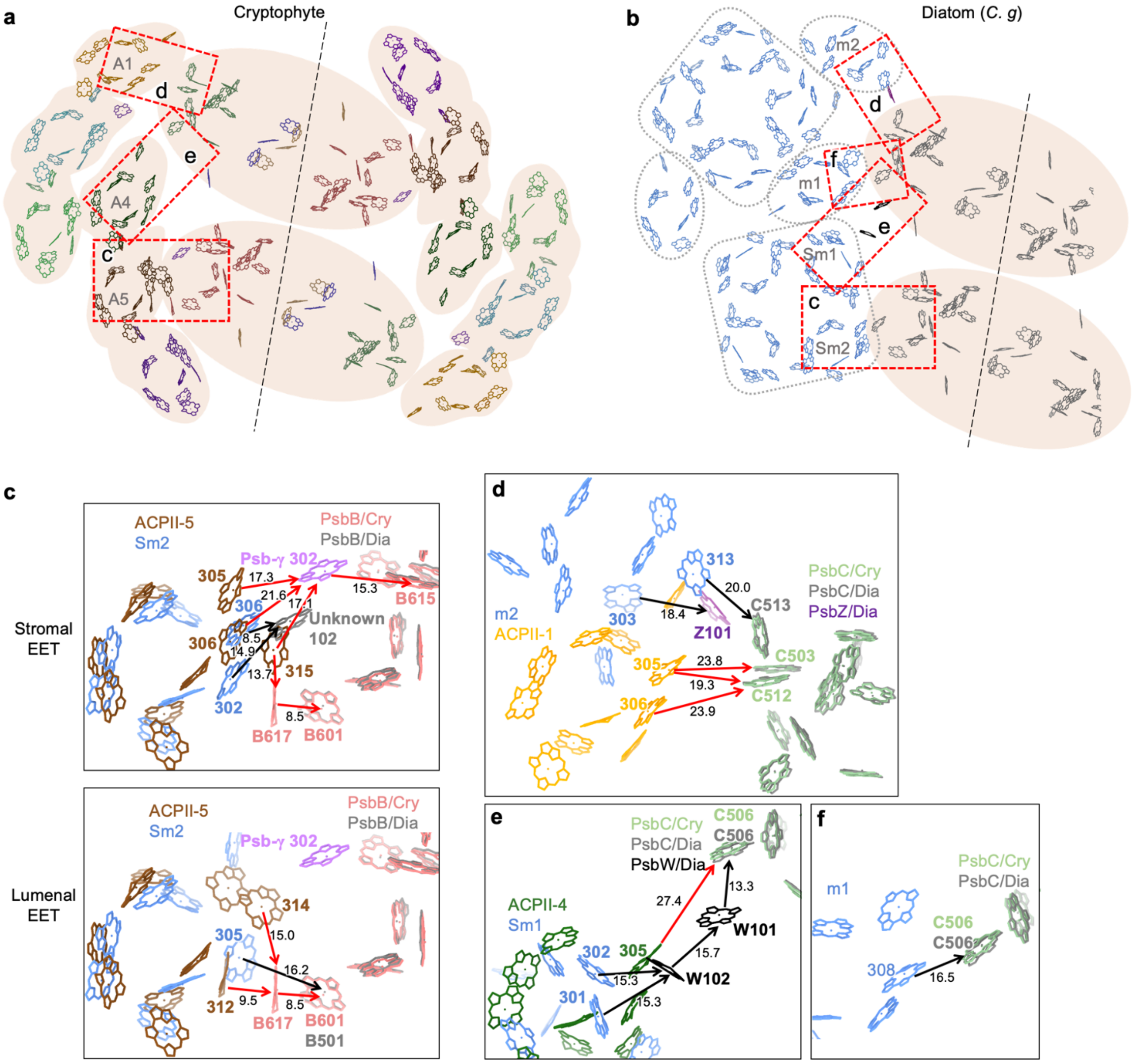

Fig. S13. Energy transfer from ACPIIs to the PSII core in cryptophyte (Cry) PSII–ACPII and comparison with those in PSII–FCPII of diatom (Dia) *Chaetoceros gracilis* (C. g). a, Chl distribution in PSII–ACPII viewed from the stromal side. Red squared areas are enlarged in panels **c-e**. **b**, Chl distribution in PSII–FCPII viewed from the stromal side. Red squared areas are enlarged in panels **c-f**. Chls in diatom FCPIIs and the PSII core are colored blue and light gray, respectively. FCPII tetramer and monomer are indicated by gray dashed square and oval, respectively. **c-f**, Comparison of the arrangements of Chls in ACPIIs and Chls of the PSII core at the interface of PCPIIs and the PSII core with those of diatom PSII–FCPII. The Mg-to-Mg distances between Chls are labeled in Å.

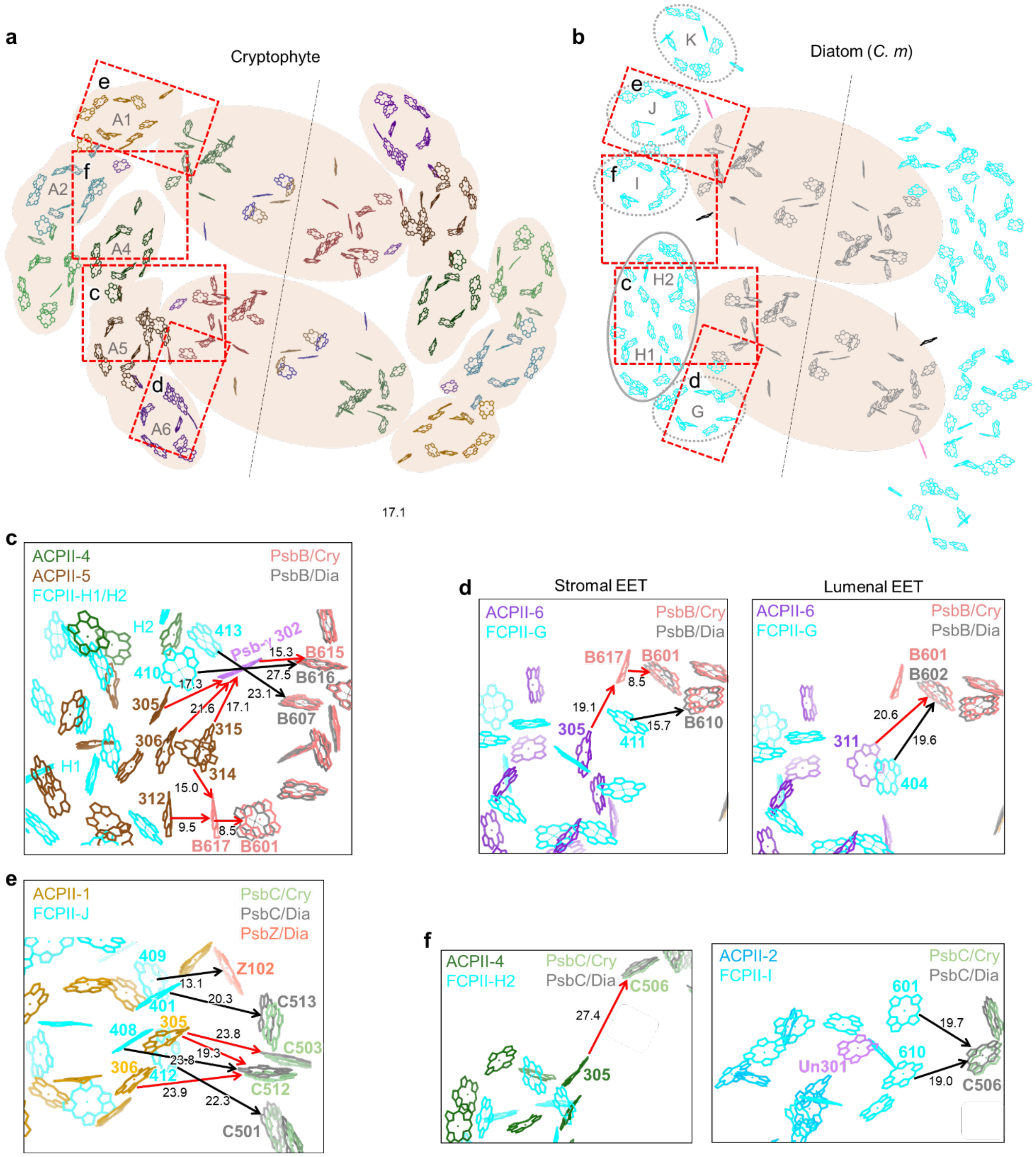

Fig. S14. Energy transfer from ACPIIs to the PSII core in cryptophyte (Cry) PSII–ACPII and comparison with those in PSII–FCPII of diatom (Dia) *Cyclotella meneghiniana* (*C. m*). **a**, Chl distribution in PSII–ACPII viewed from the stromal side. Red squared areas are enlarged in panels **c**-**f**. **b**, Chl distribution in PSII–FCPII viewed from the stromal side. Red squared areas are enlarged in panels **c**-**f**. Chls in FCPIIs and the PSII core are colored cyan and light gray, respectively. FCPII dimer and monomer are indicated by gray solid oval and dashed oval, respectively. **c**-**f**, Comparison of the arrangements of Chls in ACPIIs and Chls of the PSII core at the interface of PCPIIs and the PSII core with those of diatom PSII–FCPII. The Mg-to-Mg distances between Chls are labeled in Å.

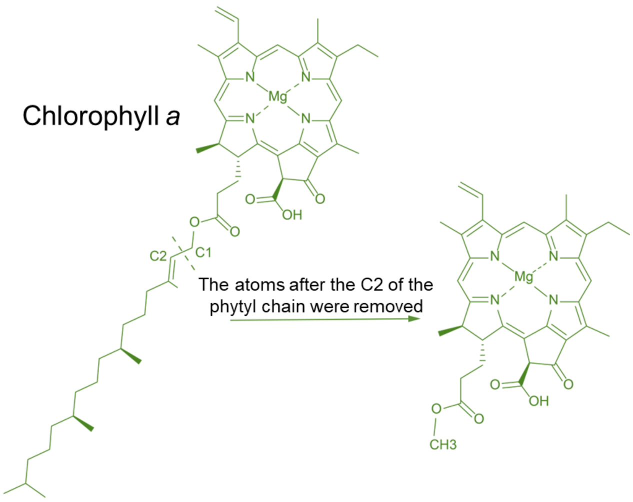

Fig. S15. The porphyrin rings of Chl molecules were selected for TDDFT (time-dependent density functional theory) calculation.
