# Supplementary material for: Structure of cryptophyte photosystem II–light-harvesting antennae supercomplex
Source: Nat Commun. 2024 Jun 12;15:4999. doi: 10.1038/s41467-024-49453-0 (PMC11169493; doi:10.1038/s41467-024-49453-0)
Supplement: Supplementary file 2 — Reporting Summary [file 41467_2024_49453_MOESM2_ESM.pdf]

nature portfolio

Corresponding author(s): Yu-Zhong Zhang, Jun Gao, Lu-Ning Liu, Long-Sheng Zhao

Last updated by author(s): Jun 5, 2024

Reporting Summary

Nature Portfolio wishes to improve the reproducibility of the work that we publish. This form provides structure for consistency and transparency in reporting. For further information on Nature Portfolio policies, see our Editorial Policies and the Editorial Policy Checklist.

Statistics

For all statistical analyses, confirm that the following items are present in the figure legend, table legend, main text, or Methods section.

n/a	Confirmed	
☐	☒	The exact sample size (n) for each experimental group/condition, given as a discrete number and unit of measurement
☐	☒	A statement on whether measurements were taken from distinct samples or whether the same sample was measured repeatedly
☒	☐	The statistical test(s) used AND whether they are one- or two-sided<br>*Only common tests should be described solely by name; describe more complex techniques in the Methods section.*
☒	☐	A description of all covariates tested
☒	☐	A description of any assumptions or corrections, such as tests of normality and adjustment for multiple comparisons
☐	☒	A full description of the statistical parameters including central tendency (e.g. means) or other basic estimates (e.g. regression coefficient) AND variation (e.g. standard deviation) or associated estimates of uncertainty (e.g. confidence intervals)
☒	☐	For null hypothesis testing, the test statistic (e.g. F, t, r) with confidence intervals, effect sizes, degrees of freedom and P value noted<br>*Give P values as exact values whenever suitable.*
☒	☐	For Bayesian analysis, information on the choice of priors and Markov chain Monte Carlo settings
☒	☐	For hierarchical and complex designs, identification of the appropriate level for tests and full reporting of outcomes
☒	☐	Estimates of effect sizes (e.g. Cohen's d, Pearson's r), indicating how they were calculated

Our web collection on statistics for biologists contains articles on many of the points above.

Software and code

Policy information about availability of computer code

Data collection	Cryo-EM Data were collected by a 300 kV Titan Krios G3i microscope (Thermo Fisher Scientific) equipped with a K3 BioQuantum direct electron detector (Gatan Inc.). Movie stacks were recorded using EPU (Thermo Fisher Scientific). Absorption spectra were measured using a Shimadzu UV–Vis 1990 spectrophotometer. Pigment composition was analyzed by LC-20AD high performance liquid chromatograph (Shimadzu, Janpan). Oxygen-evolving activity was measured by a Clark-type oxygen electrode Chlorolab2+ (Hansatech, UK). The cDNA library preparations were sequenced on an Illumina HiSeq 2000 platform.
Data analysis	ESPript 3.0, CLC Sequence Viewer 8, MUSCLE, MEGA X, cryoSPARC 3.3.1, UCSF ChimeraX, WinCOOT 0.9.8.1, PHENIX 1.20, PyMOL, Gaussian16 software, Custom python scripts

For manuscripts utilizing custom algorithms or software that are central to the research but not yet described in published literature, software must be made available to editors and reviewers. We strongly encourage code deposition in a community repository (e.g. GitHub). See the Nature Portfolio guidelines for submitting code & software for further information.

Data

Policy information about availability of data

All manuscripts must include a data availability statement. This statement should provide the following information, where applicable:

- Accession codes, unique identifiers, or web links for publicly available datasets
- A description of any restrictions on data availability
- For clinical datasets or third party data, please ensure that the statement adheres to our policy

The cryo-EM map and atomic coordinates have been deposited in the Protein Data Bank and the Electron Microscopy Data Bank under the accession numbers of 8XR6 [https://www.rcsb.org/structure/8XR6] and EMD-38596 [https://www.ebi.ac.uk/emdb/EMD-38596], respectively. The atomic coordinates data used in this study are available in the Protein Data Bank database under accession code 4YUU [https://doi.org/10.2210/pdb4YUU/pdb], 6KAF [https://doi.org/10.2210/pdb6KAF/pdb], 6JLU [https://doi.org/10.2210/pdb6JLU/pdb], 7VD5 [https://doi.org/10.2210/pdb7VD5/pdb], 7Y5E [https://doi.org/10.2210/pdb7Y5E/pdb], 7Y7B [https://doi.org/10.2210/pdb7Y7B/pdb], 8IR5 [https://doi.org/10.2210/pdb8IR5/pdb], 8J5K[https://doi.org/10.2210/pdb8J5K/pdb], 8IWH [https://doi.org/10.2210/pdb8IWH/pdb]. The RNA-seq data have been deposited in the NCBI Sequence Read Archive (SRA) database under the accession code PRJNA1120208 [https://www.ncbi.nlm.nih.gov/sra/PRJNA1120208]. Source data for Supplementary Fig. 1b, 1c, and 1d are provided in the Source Data file. Source data are provided with this paper. The custom Python scripts used in this study are available in GitHub [https://doi.org/10.5281/zenodo.10791187].

Research involving human participants, their data, or biological material

Policy information about studies with human participants or human data. See also policy information about sex, gender (identity/presentation), and sexual orientation and race, ethnicity and racism.

Reporting on sex and gender	N/A
Reporting on race, ethnicity, or other socially relevant groupings	N/A
Population characteristics	N/A
Recruitment	N/A
Ethics oversight	N/A

Note that full information on the approval of the study protocol must also be provided in the manuscript.

Field-specific reporting

Please select the one below that is the best fit for your research. If you are not sure, read the appropriate sections before making your selection.

[x] Life sciences [ ] Behavioural & social sciences [ ] Ecological, evolutionary & environmental sciences

For a reference copy of the document with all sections, see nature.com/documents/nr-reporting-summary-flat.pdf

Life sciences study design

All studies must disclose on these points even when the disclosure is negative.

Sample size	Three independent samples were used for the oxygen-evolving activity analysis. Five independent samples were used for the absorption spectra and pigment analysis. Five batches of samples were analyzed by SDS-PAGE. Similar results were obtained from the replicates of each experimental analysis with good biological reproducibility. A total of 5382 movies were recorded. 1,264,152 protein particles were picked for further process. Finally, 168,683 protein particles were selected for refinement.During the image processing, only the movies and particles with high quality and resolution were used for map construction. According to our previous knowledge on data processing and the data processing of published structures of photosystems, 168,683 protein particles with high quality and resolution are sufficient for building a map of photosystem with atomic resolution. No statistical methods were used to predetermine sample size.
Data exclusions	A fraction of the low quality and low resolution cryo-EM movies and particles were discarded.
Replication	The purification and characterization of PSI-PCPI (SDS-PAGE, absorption spectrum, pigment analysis, and oxygen-evolving activity) have been repeated independently for more than three times. Similar results were obtained from the replicates of each experimental analysis.
Randomization	Randomization is not relevant to our study as the goal of this study is to solve the structure of the specific protein supercomplex.
Blinding	Blinding is not relevant to our study because we are studying a specific protein complex. There was no existing protein structure to refer to and the data analysis was ab initio.

Reporting for specific materials, systems and methods

We require information from authors about some types of materials, experimental systems and methods used in many studies. Here, indicate whether each material, system or method listed is relevant to your study. If you are not sure if a list item applies to your research, read the appropriate section before selecting a response.

Materials & experimental systems

n/a	Involved in the study
☒	☐ Antibodies
☒	☐ Eukaryotic cell lines
☒	☐ Palaeontology and archaeology
☒	☐ Animals and other organisms
☒	☐ Clinical data
☒	☐ Dual use research of concern
☒	☐ Plants

Methods

n/a	Involved in the study
☒	☐ ChIP-seq
☒	☐ Flow cytometry
☒	☐ MRI-based neuroimaging

Plants

Seed stocks	N/A
Novel plant genotypes	N/A
Authentication	N/A
