# Supplementary material for: Structure of cryptophyte photosystem II–light-harvesting antennae supercomplex
Source: Nat Commun. 2024 Jun 12;15:4999. doi: 10.1038/s41467-024-49453-0 (PMC11169493; doi:10.1038/s41467-024-49453-0)
Supplement: Supplementary file 4 — Description of Additional Supplementary Files [file 41467_2024_49453_MOESM4_ESM.pdf]

Supplementary Data 1:

Peptides of subunits in cryptophyte PSIIACPII identified by mass spectrometry
